# Attitudes of healthcare providers towards fertility preservation in Portugal: perceived support across indications and their ethical implications

**DOI:** 10.1186/s12910-026-01477-5

**Published:** 2026-05-20

**Authors:** Alla Tirsina, Susana Silva, Cláudia de Freitas

**Affiliations:** 1 EPIUnit ITR, Institute of Public Health of the University Porto, University of Porto, Rua das Taipas, n° 135, Porto, 4050-600 Portugal; 2 Department of Public Health and Forensic Sciences, and Medical Education, Faculty of Medicine of the University of Porto, University of Porto, Alameda Prof. Hernâni Monteiro, Porto, 4200-319 Portugal; 3 Department of Sociology, Institute of Social Sciences, University of Minho, Campus de Gualtar, Braga, 4710-057 Portugal; 4 Centre for Research in Anthropology (CRIA-UMinho/IN2PAST), University of Minho, Campus de Gualtar, Braga, 4710-057 Portugal; 5 Center for Psychology at University of Porto (CPUP), Rua Alfredo Allen, Porto, 4200-135 Portugal; 6 Faculty of Psychology and Education Sciences, University of Porto, Rua Alfredo Allen, Porto, 4200-135 Portugal

**Keywords:** Fertility preservation, Attitudes, Healthcare providers, Portugal, Career development, Gender transition, Active military service or deployment.

## Abstract

**Background:**

Healthcare providers (HCPs) play a pivotal role in shaping access to and ethical deliberation around fertility preservation. While fertility preservation due to oncological and non-oncological diseases is widely accepted, its use for other reasons, such as career development, gender transition, or military deployment, raises ethical and professional questions. This study aimed to explore the attitudes of HCPs in Portugal towards fertility preservation across different indications and to identify professional factors associated with variations in perceived support.

**Methods:**

We conducted a cross-sectional observational study using an anonymous online questionnaire administered via Lime Survey between October 2024 and March 2025. A total of 298 HCPs working in Portugal were recruited through judgment sampling. Data on HCPs’ level of agreement with the use of fertility preservation for eight indications, as well as on their sociodemographic and professional characteristics, were collected. Descriptive analyses and bivariate comparisons were performed to explore levels of support and associated factors.

**Results:**

Support for fertility preservation was highest for cancer (96.3%), infertility (93.3%), benign diseases (91.3%), prevention of genetic diseases (86.3%), and age-related fertility loss (79.8%). In contrast, lower levels of support were observed for gender transition (50.7%), career development (44.3%), and active military service or deployment (41.9%), with approximately one-third of participants expressing neither agree nor disagree positions. Attitudes towards these ethically more contested indications were significantly associated with professional characteristics, including involvement with peers and patients, occupation group, working setting, and years of experience.

**Conclusion:**

HCPs in Portugal show strong support for fertility preservation in diseases contexts but reveal ambivalence regarding its use for gender transition, career development, and military purposes. This variability reflects ongoing attitudinal uncertainty and suggests that professional experience and clinical context may influence ethical positioning. Targeted educational and ethical reflection initiatives may help support HCPs in addressing the ethical, social, and technical complexities associated with fertility preservation across different contexts.

**Supplementary Information:**

The online version contains supplementary material available at 10.1186/s12910-026-01477-5.

## Introduction

The popularity of fertility preservation (i.e. a method of providing future reproductive opportunities by cryopreserving reproductive cells or tissues) has increased over the past 20 years in response to declining fertility rates [1], a growing burden of actual and anticipated infertility [2] and reduced access to reproductive healthcare services [3, 4]. Fertility preservation can be provided to people whose fertility may be compromised by serious diseases [5, 6], and in particular cancer [7, 8] and patients with endometriosis [9, 10], to transgender and non-binary people [11, 12], and to women and men who fear age-related fertility loss [13, 14] as they postpone childbearing for different reasons including financial hardship, wanting to co-parent but not having a partner, the pursuit of a career or education [15, 16], or active duty military service and deployment [17, 18].

Fertility preservation is often described as a technology of futuristic hope for fertility extension [19]. Its potential to postpone childrearing enables greater reproductive control, and choice, over the timing and practice of genetic parenthood as life unfolds according to one’s particular circumstances of family building [20–24]. While fertility preservation carries out risks, these are commonly left on the sidelines. In contrast, normative societal expectations of biogenetic childbearing, gender equality, and responsible and active ageing tend to be prioritised in discussion forums [17, 22, 25, 26]. The strong focus put onto the safety, efficacy, and cost-effectiveness of fertility preservation [2, 25], coupled with the tendency to overlook its perils, may thus be hushing the debate on contentious issues, namely: the existence of gendered, classed and racialised effects [2, 27, 28]; distributive injustice and unequal access due to differences in laws, referral policies, funding models and fertility preservation resources [4, 29, 30]; misrepresentation of the costs and success rates of the technology [17, 21]; commercialization, exploitation and financialization of fertility [31, 32]; the undermining of individual autonomy due to overreliance on the technology without the assurance of future genetic parenthood [17, 31]; health threats such as poor suboptimal pregnancy outcomes [26, 33] and emotional risks [2, 20]; and, the idealisation of the right time to give birth [3, 34].

Healthcare providers (HCPs) play a pivotal role in shaping how access to, and ethical deliberation around, fertility preservation present in society, particularly among potential users [35]. They function as gatekeepers, overseeing access to and the use of fertility preservation by a diverse range of potential users [36]. However, there is limited knowledge about HCPs’ attitudes towards fertility preservation when it is used for indications aside from imminent gonadotoxic therapy such as career development, gender transition or military deployment. A recent systematic review of quantitative evidence on HCPs’ awareness and attitudes towards fertility preservation highlights the need to broaden our understanding of their views, especially among professionals other than oncology specialists and beyond topics related to female fertility [37]. This review emphasizes the importance of exploring opinions about a wider array of fertility preservation indications. Moreover, most existing studies to date have been conducted in countries like the United States of America, China, the Netherlands, France, India, Iran, Japan, Turkey, Canada, Indonesia and the United Kingdom [37]. Expanding this geographic focus is necessary to strengthen the evidence base on Southern European countries such as Portugal, where the interplay between public and private healthcare provision, the persistence of patriarchal family norms, and negative attitudes towards “unconventional” paths to parenthood may shape access to fertility preservation [38]. Examining HCPs’ attitudes towards fertility preservation in Portugal across different indications may also inform the development of context-sensitive European guidelines that account for social, ethical, and professional diversity. Furthermore, incorporating the views of HCPs into the broader European debate will contribute to ongoing ethical discussions on reproductive autonomy, distributive justice, and the scope of medical responsibility in the provision of fertility preservation [39, 40].

Thus, this study aimed to explore the attitudes of HCPs working in Portugal towards fertility preservation across different indications and to identify professional factors associated with variations in perceived support.

## Methods

### Study design and data collection

This cross-sectional observational study is based on an online questionnaire, which was available between October 2024 and March 2025 in LimeSurvey. The questionnaire was designed by the research team following a thorough review of the literature and of an exhaustive inventory of existing questionnaires about awareness, knowledge and attitudes of HCPs towards fertility preservation [15, 41, 42]. The final questionnaire is available as Supplementary Material 1.

Participation by HCPs working in Portugal but who were not enrolled in educational programmes (e.g., undergraduate students, interns, residents and fellows) was first requested via the mailing lists of fifteen relevant professional associations and societies related to reproductive medicine and twenty-six assisted reproduction centres. Of the 41 institutions invited, 34 agreed to disseminate the questionnaire. Four months later, the authors also asked their own colleagues to distribute the questionnaire among their networks. This judgement sample was the most convenient and economical approach while also being technically and conceptually appropriate. A total of 302 questionnaires were completed.

This article focuses on data relating to HCPs’ attitudes towards fertility preservation according to the following sociodemographic and professional characteristics: gender (female, male, another gender, or prefer not to answer); age; level of education; occupational group; occupation; healthcare specialty group; working environment (categorised as public institutions, private institutions, or both); working experience (in years); and involvement with peers and patients. With regard to their involvement with peers and patients, participants were asked to report how often the following situations occurred over the past year, categorising their responses as *always/often*,* sometimes*,* or rarely/never: Have you referred someone to a specialist in medically assisted reproduction? Have you asked a colleague who is an expert in this field about fertility preservation? Have you talked to your patients about fertility preservation? Have you provided your patients with oral and/or written information about fertility preservation?*

The occupational group of the HCPs (categorised as healthcare professionals and professors, directors and executive managers, intermediate-level technicians and life science related occupations, and personal care workers who provide personal care services directly to patients in healthcare and residential settings) and their occupation (categorised as medical doctor, nurse, embryologist or biologist, and other) were coded according to the Portuguese Classification of Occupations [43] and the World Health Organization classification of health workers [44]. The professional specialty was evaluated using an open-ended item. The two first authors independently grouped specialties into six categories established a posteriori (Obstetrics and Gynaecology, General/Primary Care, Reproductive Medicine, Paediatrics and Child Health, Diagnostic/Laboratory specialties, and Other) and disagreements in classification were resolved by consensus.

Participants were asked to indicate their level of agreement with the use of fertility preservation for various indications, categorised as follows: cancer; infertility; benign diseases such as endometriosis, lupus, or Turner syndrome; prevention of transmissible genetic diseases such as Huntington’s disease (where individuals may elect to postpone childbearing due to uncertainty about their genetic status); age related fertility loss; gender transition; development of a professional career; and, active military service or deployment. To assess the associations between the variables, and to capture the substantively meaningful threshold aligned with the study’s research question (support and non-support), these answers were dichotomised into agree (including *strongly agree* and *agree*) and other (including *neither agree nor disagree*, *disagree* and *strongly disagree*).

Following the exclusion of four participants (one with missing values for the outcome variables and three not eligible, comprising two residents and one student), 298 HCPs were included in the quantitative analysis.

### Ethical aspects

Ethical approval was granted by the Ethics Committee of the Institute of Public Health of the University of Porto and the Data Protection Officer (process n.o CE23240). Online informed consent was obtained from all HCPs prior to participation in the study. The management of data was conducted to ensure confidentiality and protection at each stage of the data collection, storage, exchange and preservation process. The research procedures were carried out in line with the principles set out in the Declaration of Helsinki (1964) and subsequent amendments.

### Data analysis

The responses are presented as counts and proportions, compared using the chi-square test or the Fisher’s exact test when appropriate. To limit data variability and avoid distorting statistical associations, the analysis of associations was not performed for items with a ceiling effect, i.e. more than 80% of participants answered *agree* or *strongly agree* to the fertility preservation indication [45]. Bivariate comparison was employed to facilitate mapping of patterns and variations, and to lay the groundwork for future hypothesis-driven studies. Statistical significance was set at a value of *p* < 0.05. Analyses were performed using the IBM Statistical Package for the Social Sciences (SPSS) Statistics for Windows, version 30.0 (Armonk, NY, USA).

## Results

The sociodemographic and professional characteristics of the participants are shown in Table 1. The vast majority were healthcare professionals and professors (90.6%), predominantly medical doctors (47.0%) and nurses (44.0%), and specialists in Obstetrics and Gynaecology (37.4%) and General/Primary Care (22.1%). Approximately one-third of the participants had less than 11 years of professional experience. Most participants were female (89.9%), under 40 years of age (52.6%), and about half held a Master’s degree (52.1%).

**Table 1:** Sociodemographic and professional characteristics of the participants

	Total *n* (%)
Gender (*n* = 297)	
Female	267 (89.9)
Male	30 (10.1)
Age, years (*n* = 297)	
21–30	37 (12.5)
31–40	119 (40.1)
41–50	88 (29.6)
> 51	53 (17.8)
Level of education (*n* = 286)	
Licentiate	121 (42.3)
Master	149 (52.1)
PhD	16 (5.6)
Occupational group (*n* = 297)	
Healthcare professionals and professors	269 (90.6)
Directors and executive managers	14 (4.7)
Intermediate-level technicians and occupations related to life sciences	9 (3.0)
Personal care workers in health services	5 (1.7)
Occupation (*n* = 298)	
Medical Doctor	140 (47.0)
Nurse	131 (44.0)
Embryologist or Biologist	16 (5.4)
Other^a^	11 (3.7)
Healthcare specialty group (*n* = 235)	
Obstetrics and Gynaecology^b^	88 (37.4)
General/Primary Care^c^	52 (22.1)
Reproductive Medicine^d^	20 (8.5)
Paediatrics and Child Health^e^	14 (6.0)
Diagnostic/Laboratory Specialties^f^	19 (8.1)
Other^g^	42 (17.9)
Working environment (*n* = 292)	
Only public institutions	85 (29.1)
Only private institutions	23 (7.9)
Both public and private institutions	184 (63.0)
Working experience, years (*n* = 297)	
≤ 10	95 (32.0)
11–15	71 (23.9)
> 15	131 (44.1)
Referred someone to a specialist in medically assisted reproduction (*n* = 297)	
Often/Always	61 (20.5)
Sometimes	96 (32.3)
Never/Rarely	140 (47.1)
Asked a colleague about fertility preservation (*n* = 297)	
Often/Always	51 (17.2)
Sometimes	111 (37.4)
Never/Rarely	135 (45.5)
Talk with patients about fertility preservation (*n* = 297)	
Often/Always	61 (20.5)
Sometimes	110 (37.0)
Never/Rarely	126 (42.4)
Provide information to patients about fertility preservation (*n* = 297)	
Often/Always	63 (21.2)
Sometimes	82 (27.6)
Never/Rarely	152 (51.2)

^a^Psychologist, technician, secretary, administrative specialist, administrative manager, pharmacist, researcher, psychotherapist, radiologist; ^b^Obstetrics and Gynaecology (medical doctors), Maternity and Gynaecology (nurses); ^c^General Practice and Family Medicine (medical doctors), Community Nursing (nurses); ^d^Reproductive medicine, Andrology; ^e^Paediatry (medical doctors), Paediatrics and child health (nurses); ^f^Clinical Embryology, Genetics, Microbiology, Molecular Biology, Nuclear Medicine (medical doctor), Radiology; ^g^Medical-surgical nursing, Rehabilitation, Oncology, Clinical psychology, Haemato-oncology, Haematology, Gerontology (nurse), Clinical secretariat, Internal medicine (medical doctor), Breast Pathology, Mental Health and Psychotherapy

^*^The total does not add up to 298 owing to non-responses

### HCPs’ levels of agreement according to the indication for fertility preservation

Table 2 presents participants’ level of agreement regarding the use of fertility preservation across various indications. Participants’ support was highest for the following indications: cancer (96.3%); infertility (93.3%); benign diseases (91.3%); prevention of transmissible genetic diseases (86.3%); and age-related fertility loss (79.8%). Lower levels of agreement were observed for gender transition (50.7%), career development (44.3%) and active military service or deployment (41.9%). Notably, approximately one-third of participants neither agreed nor disagreed with the use of fertility preservation in the latter indications.

**Table 2:** Participants’ levels of agreement regarding the use of fertility preservation across various indications (*N* = 298)

Indications for using fertility preservation	Strongly disagree	Disagree	Neither agree nor disagree	Agree	Strongly agree
Cancer, *n (%)*	0 (0.0)	3 (1.0)	8 (2.7)	60 (20.1)	227 (76.2)
Infertility, *n (%)*	2 (0.7)	5 (1.7)	13 (4.4)	78 (26.2)	200 (67.1)
Benign diseases, *n (%)*	0 (0.0)	5 (1.7)	21 (7.0)	96 (32.2)	176 (59.1)
Prevention of transmissible genetic diseases, *n (%)*	6 (2.0)	12 (4.0)	23 (7.7)	84 (28.2)	173 (58.1)
Age-related fertility loss, *n (%)*	3 (1.0)	21 (7.0)	36 (12.1)	113 (37.9)	125 (41.9)
Gender transition, *n (%)*	22 (7.4)	20 (6.7)	105 (35.2)	82 (27.5)	69 (23.2)
Career development, *n (%)*	20 (6.7)	48 (16.1)	98 (32.9)	82 (27.5)	50 (16.8)
Military service or deployment, *n (%)*	20 (6.7)	40 (13.4)	113 (37.9)	79 (26.5)	46 (15.4)

The proportions may not add 100 due to rounding

### Factors influencing HCPs’ attitudes towards fertility preservation

HCPs who were more often involved in discussions about fertility preservation with their peers and who talked about it and provided information to their patients were significantly more likely to support its use for gender transition, career development, and active military service/deployment as shown in Table 3. Support for fertility preservation in the context of gender transition was lower among participants with more than 15 years of experience. Healthcare professionals and professors were significantly less likely to support the use of fertility preservation for career-related and military reasons compared to other occupational groups. Conversely, those working exclusively in private institutions showed higher acceptance of fertility preservation for career development purposes, as opposed to those working only in the public sector or both in public and private institutions. 

**Table 3:** Healthcare providers’ opinions on fertility preservation across non-medical indications, by professional and sociodemographic characteristics

		Gender transition		Career development		Military service or deployment	
	Total	Agree	Other^a^	Agree	Other	Agree	Other
		*n (%)*	*n (%)*	*n (%)*	*n (%)*	*n (%)*	*n (%)*
Gender							
Female	267	137 (51.3)	130 (48.7)	116 (43.4)	151 (56.6)	112 (41.9)	155 (58.1)
Male	30	14 (46.7)	16 (53.3)	16 (53.3)	14 (46.7)	13 (43.3)	17 (56.7)
Age (years)							
21–30	37	20 (54.1)	17 (45.9)	19 (51.4)	18 (48.6)	14 (37.8)	23 (62.2)
31–40	119	65 (54.6)	54 (45.4)	51 (42.9)	68 (57.1)	47 (39.5)	72 (60.5)
41–50	88	45 (51.1)	43 (48.9)	40 (45.5)	48 (54.5)	37 (42.0)	51 (58.0)
> 51	53	21 (39.6)	32 (60.4)	22 (41.5)	31 (58.5)	27 (50.9)	26 (49.1)
Level of education							
Licentiate	121	62 (51.2)	59 (48.8)	48 (39.7)	73 (60.3)	49 (40.5)	72 (59.5)
Master	149	75 (50.3)	74 (49.7)	69 (46.3)	80 (53.7)	61 (40.9)	88 (59.1)
PhD	16	11 (68.8)	5 (31.3)	11 (68.8)	5 (31.3)	8 (50.0)	8 (50.0)
Occupational group							
Healthcare professionals and professors	269	135 (50.2)	134 (49.8)	112 (41.6)*	157 (58.4)*	108 (40.1)*	161 (59.9)*
Other^b^	28	16 (57.1)	12 (42.9)	20 (71.4)*	8 (28.6)*	17 (60.7)*	11 (39.3)*
Occupation							
Medical Doctor	140	77 (55.0)	63 (45.0)	59 (42.1)	81 (57.9)	55 (39.3)	85 (60.7)
Nurse	131	60 (45.8)	71 (54.2)	56 (42.7)	75 (57.3)	54 (41.2)	77 (58.8)
Other^c^	27	14 (51.9)	13 (48.1)	17 (63.0)	10 (37.0)	16 (59.3)	11 (40.7)
Healthcare specialty group							
Obstetrics and Gynaecology^d^	88	43 (48.9)	45 (51.1)	36 (40.9)	52 (59.1)	34 (38.6)	54 (61.4)
General/Primary Care^e^	52	26 (50.0)	26 (50.0)	18 (34.6)	34 (65.4)	20 (38.5)	32 (61.5)
Reproductive Medicine^f^	20	15 (75.0)	5 (25.0)	14 (70.0)	6 (30.0)	11 (55.0)	9 (45.0)
Paediatrics and Child Health^g^	14	4 (28.6)	10 (71.4)	6 (42.9)	8 (57.1)	3 (21.4)	11 (78.6)
Diagnostic/Laboratory Specialties^h^	19	10 (52.6)	9 (47.4)	9 (47.4)	10 (52.6)	10 (52.6)	9 (47.4)
Other^i^	42	22 (52.4)	20 (47.6)	23 (54.8)	19 (45.2)	22 (52.4)	20 (47.6)
Working environment							
Only public institutions	85	37 (43.5)	48 (56.5)	33 (38.8)*	52 (61.2)*	32 (37.6)	53 (62.4)
Only private institutions	23	15 (65.2)	8 (34.8)	17 (73.9)*	6 (26.1)*	14 (60.9)	9 (39.1)
Both public and private institutions	184	98 (53.3)	86 (46.7)	81 (44.0)*	103 (56.0)*	78 (42.4)	106 (57.6)
Working experience (years)							
≤ 10	95	50 (52.6)*	45 (47.4)*	45 (47.4)	50 (52.6)	38 (40.0)	57 (60.0)
11–15	71	44 (62.0)*	27 (38.0)*	29 (40.8)	42 (59.2)	28 (39.4)	43 (60.6)
> 15	131	57 (43.5)*	74 (56.5)*	58 (44.3)	73 (55.7)	59 (45.0)	72 (55.0)
Referred someone to a specialist in medically assisted reproduction							
Often/Always	61	38 (62.3)	23 (37.7)	33 (54.1)	28 (45.9)	27 (44.3)	34 (55.7)
Sometimes	96	46 (47.9)	50 (52.1)	40 (41.7)	56 (58.3)	43 (44.8)	53 (55.2)
Never/Rarely	140	66 (47.1)	74 (52.9)	58 (41.4)	82 (58.6)	55 (39.3)	85 (60.7)
Asked a colleague about fertility preservation							
Often/Always	51	38 (74.5)*	13 (25.5)*	32 (62.7)*	19 (37.3)*	29 (56.9)*	22 (43.1)*
Sometimes	111	54 (48.6)*	57 (51.4)*	51 (45.9)*	60 (54.1)*	48 (43.2)*	63 (56.8)*
Never/Rarely	135	58 (43.0)*	77 (57.0)*	48 (35.6)*	87 (64.4)*	48 (35.6)*	87 (64.4)*
Talk with patients about fertility preservation							
Often/Always	61	40 (65.6)*	21 (34.4)*	38 (62.3)*	23 (37.7)*	32 (52.5)	29 (47.5)
Sometimes	110	54 (49.1)*	56 (50.9)*	47 (42.7)*	63 (57.3)*	47 (42.7)	63 (57.3)
Never/Rarely	126	56 (44.4)*	70 (55.6)*	46 (36.5)*	80 (63.5)*	46 (36.5)	80 (63.5)
Provide information to patients about fertility preservation							
Often/Always	63	43 (68.3)*	20 (31.7)*	39 (61.9)*	24 (38.1)*	35 (55.6)*	28 (44.4)*
Sometimes	82	40 (48.8)*	42 (51.2)*	40 (48.8)*	42 (51.2)*	36 (43.9)*	46 (56.1)*
Never/Rarely	152	67 (44.1)*	85 (55.9)*	52 (34.2)*	100 (65.8)*	54 (35.5)*	98 (64.5)*

The total may not add up to 298 participants in each variable due to missing values; * *p* < 0.05; ^a^ Participants who answered “neither agree nor disagree”, “disagree” and “strongly disagree”; ^b^ Directors and executive managers, intermediate-level technicians and occupations related to life sciences, and personal care workers in health services; ^c^ Embryologist, biologist, psychologist, technician, secretary, administrative specialist, administrative manager, pharmacist, researcher, psychotherapist, radiologist; ^d^ Obstetrics and Gynaecology (medical doctors), Maternity and Gynaecology (nurses); ^e^ General Practice and Family Medicine (medical doctors), Community Nursing (nurses); ^f^ Reproductive medicine, Andrology; ^g^ Paediatrics (medical doctors), Paediatrics and child health (nurses); ^h^ Clinical Embryology, Genetics, Microbiology, Molecular Biology, Nuclear Medicine (medical doctor) and Radiology; ^i^ Medical-surgical nursing, Rehabilitation (nurse), Oncology, Clinical psychology, Haemato-oncology, Haematology, Gerontology (nurse), Clinical secretariat, Internal medicine (medical doctor), Breast Pathology, Mental Health and Psychotherapy

## Discussion

This study uncovered a nuanced spectrum of attitudes among HCPs in Portugal towards fertility preservation, characterised by strong consensus regarding indications related to medical diseases and high ambivalence regarding its use for other purposes. HCPs overwhelmingly supported fertility preservation when tied to cancer, infertility, and benign or transmissible genetic diseases. However, they expressed diverse positions in the contexts of gender transition, career development, and military service/deployment — areas that remain understudied and carry ethical and practical complexities. The results suggest that attitudes towards the latter indications tend to be influenced by factors related to professional experiences, rather than other sociodemographic characteristics. Engagement in information exchange about fertility preservation with peers and patients is associated with favourable attitudes towards its use in gender transition, career development or military contexts. Working solely in the private sector was also positively associated with support for fertility preservation for career advancement. While healthcare professionals and professors were less supportive regarding career- and military-related uses than other occupational groups, the HCPs with over 15 years of working experience were less supportive in the context of gender transition.

This study is novel in exploring and comparing HCPs’ attitudes towards fertility preservation across a broad range of indications, extending beyond the predominant focus on oncological diseases and the views of oncologists observed in previous research [37, 46–49]. The inclusion of general practitioners (GPs), family physicians and nurses from various backgrounds, and other HCPs strengthens its contribution. Methodologically, it also avoids the commonly used convenient-sampling [16, 31], representing the first study in Portugal to evaluate attitudes towards fertility preservation for non-cancer related reasons. This research adds crucial insights to the Southern European debate, particularly within the Portuguese healthcare system, which faces specific constraints affecting access to fertility preservation, including resources limitations, age-related policies, and partial privatisation [50, 51]. Moreover, this study provides baseline evidence prior to recent legislative changes expanding public coverage of fertility preservation for certain benign conditions in Portugal, namely endometriosis and adenomyosis [52]. As such, it offers insight into professional attitudes at a transitional moment, with implications for the ethical evaluation and implementation of emerging fertility preservation policies.

Our findings reveal strong support for fertility preservation in the context of cancer, infertility, benign diseases, and genetic disease prevention, which mirrors existing literature [49, 53]. Conversely, support wanes for gender transition, career planning, and military deployment, which are more often perceived as elective rather than medically necessary [7, 34, 54–56]. This distinction reflects differences in the perceived legitimacy of, and autonomy over, decision-making processes guided by the primacy of medical justifications over socially-driven needs and preferences [26, 54–56]. Attitudes towards age-related fertility loss fall somewhere in between, reflecting its growing biomedicalisation, i.e. the framing of a natural process as a condition requiring medical intervention [3, 29].

One-third of all the study participants expressed neither agree nor disagree positions about fertility preservation for gender transition, career, and military reasons. Selecting such a response may be indicative of participants’ ambivalence, conflicted feelings, or apathy. It may partly reflect insufficient awareness and knowledge, as documented in earlier studies [42, 57–59], suggesting the importance of educational and reflective initiatives at the organisational level [37]. Such initiatives may enhance knowledge, promote involvement in fertility-related discussions, and support more informed decision-making [60–62]. Neither agree nor disagree positioning in the context of gender transition may also reflect tensions between evolving frameworks of reproductive justice and more traditional models of medical gatekeeping. Although access to medically assisted reproduction independent of gender identity has been granted in 2016 in Portugal, its implementation remains under-explored [40, 63]. Neither agree nor disagree regarding career-related fertility preservation may also reveal ethical concerns about potential social or institutional pressures to delay parenthood [64, 65]. Differences between public and private sector professionals suggest that institutional context, including resource allocation considerations and organisational cultures, may shape these attitudes [66, 67]. Regarding military purposes, this uncertainty may be indicative of social distance or disconnection, given that the utilisation of fertility preservation for such purposes is rarely discussed in Portugal.

More experienced HCPs were less supportive of fertility preservation for gender transition, which may reflect lower exposure to recent developments in care practices. As mentioned above, access to assisted reproduction independently of gender identity was established recently [68], and specialised gender transition care has expanded since 2018 [63]. These relatively recent changes may contribute to generational differences in familiarity and acceptance.

Frequent involvement with fertility preservation in clinical practice through discussions with colleagues or patients and the use of informational resources was associated with supporting its use for gender transition, career, and military service reasons. These results underscore the role of informal learning, clinical exposure and ethical reflection in shaping professional attitudes [69]. However, barriers such as time constraints, insufficient staff, and lack of institutional support may hinder these processes, as documented in other settings [70–72]. Addressing such barriers in the Portuguese context may help foster more informed and consistent approaches to fertility preservation.

Although not statistically significant, differences across healthcare specialities are noteworthy. Specialists in reproductive medicine showed greater support for fertility preservation in contexts of gender transition, career development and, to a lesser extent, military service or deployment, possibly reflecting both their clinical exposure and sensitivity to diverse patient needs. In contrast, specialists in paediatrics and child health were less supportive, especially for gender transition and military purposes. This may be related to their more limited engagement with fertility preservation outside of disease contexts, as well as its still evolving evidence base in paediatric populations [73–79].

This study has several limitations. The low participation of psychologists and reproductive medicine specialists may limit generalizability. The sample is also skewed towards female respondents and certain professional groups, introducing potential selection bias. Additionally, elective fertility preservation was only examined in relation to career development, excluding other relevant motivations. Participants were also not asked to classify their views along ideological lines.

Further research integrating multivariable analyses and qualitative methodologies would deepen understanding of these findings. While multivariable approaches could identify independent predictors of attitudes, qualitative studies would provide more nuanced insight into the underlying values, experiences, and contextual factors shaping HCPs’ attitudes. Such approaches are vital for informing future research, policy development, and clinical practice in fertility preservation.

## Conclusion

HCPs in Portugal show strong support for the use of fertility preservation due to oncological and non-oncological diseases and considerable ambivalence regarding its use for gender transition, career development and military deployment. Attitudes towards age-related fertility loss falls in between the two ends of the spectrum. The findings provide insights on how ethical distinctions between necessity and choice may influence professional positionings. They highlight the role of professional experiences, suggesting that informal exchange of information, clinical exposure and ethical reflection shape HCP’s attitudes towards its use for indications beyond oncological and non-oncological diseases.

Our results call for a twofold strategy to address the ethical, social, and technical issues associated with the use of fertility preservation. On the one hand, it is necessary to continue promoting access to formal education and ethical reflection forums, where HCPs can engage with questions of reproductive autonomy, distributive justice and gender equality. On the other hand, it is crucial to support informal learning and ethical reflection cultures within healthcare settings that can facilitate the exchange of information, as well as of concerns and peer support. Special attention should be given to primary care providers, especially GPs and family physicians, who frequently serve as gatekeepers in reproductive counselling and care.

## Supplementary Information

Supplementary Material 1: Supplementary Material 1 – Online Questionnaire.

## Data Availability

All data generated or analysed during this study are included in this published article [and its supplementary information files].

## Abbreviations

HCPs: Healthcare providers
