# Supplementary material for: Attitudes of healthcare providers towards fertility preservation in Portugal: perceived support across indications and their ethical implications
Source: BMC Med Ethics. 2026 May 20;27:133. doi: 10.1186/s12910-026-01477-5 (PMC13378160; doi:10.1186/s12910-026-01477-5)
Supplement: Supplementary file 1 — Supplementary Material 1: Supplementary Material 1 – Online Questionnaire. [file 12910_2026_1477_MOESM1_ESM.docx]

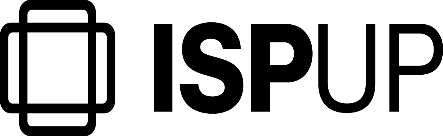

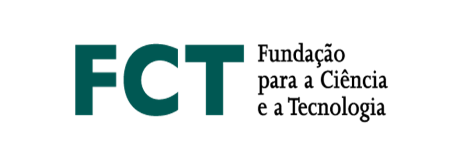

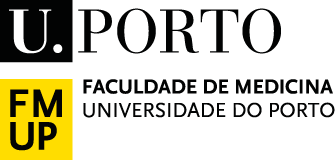


**QUESTIONNAIRE**

***“Fertility preservation in Portugal: awareness and attitudes of healthcare providers”***

(FCT PhD grant Ref. SFRH/BD/05232/2021, DOI 10.54499/2021.05232.BD)

This study aims to assess awareness and attitudes of healthcare providers toward fertility preservation in Portugal.

*There are no right or wrong answers. We are interested in knowing your honest opinion.* Thank you in

advance for your collaboration and for taking the time to answer this questionnaire.

*The duration required for completion: 5 minutes****.***

***Section I.*** AWARENESS OF FERTILITY PRESERVATION

1. How do you feel regarding the information you have about fertility preservation?

| Not informed at all |  |  |  | Highly informed |
| --- | --- | --- | --- | --- |
| □ | □ | □ | □ | □ |

1. Please state the **two main** sources where do you seek information on fertility preservation:

Healthcare professionals and/or professional networks □

Scientific papers (including directives) □

Printed Media (Newspapers, Magazines) □ Broadcast Media (TV, Radio) □

Digital Media (Apps, Emails, Social media - Twitter, LinkedIn, Instagram, YouTube) □

Out-of-home Media (Billboards, Ads at public transport stations) □

Academic education (licentiate, master, etc.) □

Friends and family □

Other □

1. How do you feel about the information you have on the following fertility preservation options?

|  | Not informed at all |  |  |  | Highly informed |
| --- | --- | --- | --- | --- | --- |
| Cryopreservation of ovarian tissue | □ | □ | □ | □ | □ |
| Cryopreservation of oocytes | □ | □ | □ | □ | □ |
| Cryopreservation of sperm | □ | □ | □ | □ | □ |
| Cryopreservation of testicular tissue | □ | □ | □ | □ | □ |
| Embryo cryopreservation | □ | □ | □ | □ | □ |
| Pre-treatment with GnRH agonists (such as depot leuprolide injections) | □ | □ | □ | □ | □ |
| Ovarian transposition (surgical treatment) | □ | □ | □ | □ | □ |
| Spermatogonial stem cells (SSCs) cryopreservation | □ | □ | □ | □ | □ |

1. During the last year, how often did you carry out the following activities:

|  | Never/Rarely | Sometimes | Often/Always |
| --- | --- | --- | --- |
| Refer the patient to a fertility specialist, reproductive endocrinologist, or specialist gynaecologist in fertility care | □ | □ | □ |
| Consult a fertility specialist, reproductive endocrinologist, or specialist gynaecologist in fertility care with questions on the fertility preservation of my patient | □ | □ | □ |
| Discuss with the patient about fertility preservation | □ | □ | □ |
| Provide oral and/or written information to the patient about fertility preservation | □ | □ | □ |

1. Do you feel the need to deepen your knowledge about fertility preservation?

Yes □ No □

If "Yes", please indicate which topics:

______________________________________________________________________

­­­______________________________________________________________________

***Section II.*** ATTITUDES TOWARDS FERTILITY PRESERVATION

1. It is possible to resort to fertility preservation for several reasons. To what extent do you agree with the use of fertility preservation in the following situations:

|  | Strongly Disagree | Disagree | Neither Agree or Disagree | Agree | Strongly Agree |
| --- | --- | --- | --- | --- | --- |
| Malignant disease (cancer) | □ | □ | □ | □ | □ |
| Infertility | □ | □ | □ | □ | □ |
| Benign disease (endometriosis, lupus, Turner Syndrome) | □ | □ | □ | □ | □ |
| Avoidance of genetic transmissible diseases | □ | □ | □ | □ | □ |
| Age related fertility loss | □ | □ | □ | □ | □ |
| Development of a professional career | □ | □ | □ | □ | □ |
| Gender transition | □ | □ | □ | □ | □ |
| Active military service or deployment | □ | □ | □ | □ | □ |

1. To what extent would you advise a relative or a close friend to resort to fertility preservation in the following situations:

|  | Never/Rarely | Sometimes | Often/Always |
| --- | --- | --- | --- |
| Malignant disease (cancer) | □ | □ | □ |
| Infertility | □ | □ | □ |
| Benign disease (endometriosis, lupus, Turner Syndrome) | □ | □ | □ |
| Avoidance of genetic transmissible diseases | □ | □ | □ |
| Age related fertility loss | □ | □ | □ |
| Development of a professional career | □ | □ | □ |
| Active military service or deployment | □ | □ | □ |

It is important for us to understand the reason(s) for your answers above. Please give a brief explanation.

____________________________________________________________________________________________________________________________________________________

***Section III.*** SOCIODEMOGRAPHIC AND SOCIO-PROFESSIONAL CHARACTERISTICS

1. What is your occupation?

Medical Doctor (MD) □

Nurse □

Embryologist □

Biologist □

Psychologist □

Other_________________

1. What is your specialty/subspecialty? __________________________________
2. At this moment, which situation best describes your professional position?

Directors and executive managers □

Healthcare professionals and Professors □

Intermediate-level technicians and occupations related to life sciences □

Personal Care Workers in Health Services □

1. How many years have you been working as a healthcare professional?

≤ 5 □

6-10 □

11-15 □

> 15 □

1. Do you have professional experience:

|  | No | Yes, just in one setting | Yes, in many settings |
| --- | --- | --- | --- |
| In public health care settings | □ | □ | □ |
| In private health care settings | □ | □ | □ |

1. What is the highest educational level you have completed?

Bachelor’s degree □

Licentiate degree □

Master’s/ Integrated Master’s □

PhD □

Other___________________________

1. How old are you?

21 – 30 years □

31 – 40 years □

41 – 50 years □

51 – 60 years □

> 61 years □

1. Gender:

Female □

Male □

Another gender □

Prefer not to answer □

***Section IV.*** COMMENTS AND/OR SUGGESTIONS

Please, provide herein your comments and/or suggestions:

__________________________________________________________________________________________________________________________________________________________________________

**Thank you for your collaboration!**
